# Fault Detection in Power Equipment via an Unmanned Aerial System Using Multi Modal Data

**DOI:** 10.3390/s19133014

**Published:** 2019-07-09

**Authors:** Bushra Jalil, Giuseppe Riccardo Leone, Massimo Martinelli, Davide Moroni, Maria Antonietta Pascali, Andrea Berton

**Affiliations:** 1 Istituto di Scienza e Tecnologie dell’Informazione “Alessandro Faedo” CNR, 56124 Pisa, Italy; 2 Istituto di Fisiologia Clinica CNR, 56124 Pisa, Italy

**Keywords:** image analysis, RGB images, infrared images, wire detection, unmanned aerial vehicles, object detection, neural networks

## Abstract

The power transmission lines are the link between power plants and the points of consumption, through substations. Most importantly, the assessment of damaged aerial power lines and rusted conductors is of extreme importance for public safety; hence, power lines and associated components must be periodically inspected to ensure a continuous supply and to identify any fault and defect. To achieve these objectives, recently, Unmanned Aerial Vehicles (UAVs) have been widely used; in fact, they provide a safe way to bring sensors close to the power transmission lines and their associated components without halting the equipment during the inspection, and reducing operational cost and risk. In this work, a drone, equipped with multi-modal sensors, captures images in the visible and infrared domain and transmits them to the ground station. We used state-of-the-art computer vision methods to highlight expected faults (i.e., hot spots) or damaged components of the electrical infrastructure (i.e., damaged insulators). Infrared imaging, which is invariant to large scale and illumination changes in the real operating environment, supported the identification of faults in power transmission lines; while a neural network is adapted and trained to detect and classify insulators from an optical video stream. We demonstrate our approach on data captured by a drone in Parma, Italy.

## 1. Introduction

**Figure 1:**
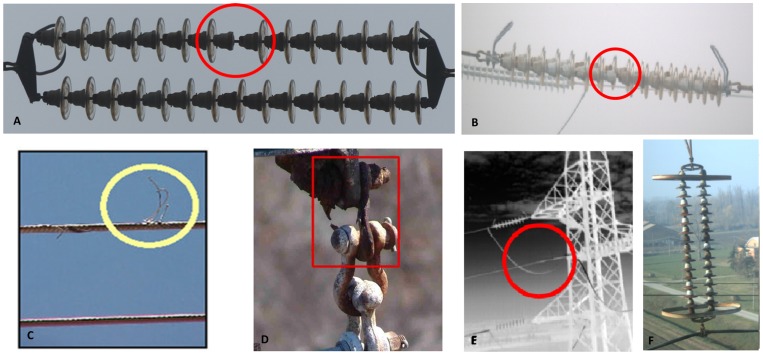
A sample of common defects: (**A**) missing plate along the insulator chain; (**B**) missing plate along the (rusted) insulator chain; (**C**) damaged strand of the cable; (**D**) hanging point, damaged by rust; (**E**) cable joints, which are more frequently affected by hot spots; (**F**) a chain of insulators, rusted.

Power transmission lines are the means of electricity distribution, and it is of extreme importance to ensure the continuous supply of electricity and the high performance of these lines. Constant surveillance and inspection of power lines can play a vital role to avoid power shortage: detection of defects in power equipment at an early stage can prevent severe and costly damage, and even used to expect future anomalies. Generally, the electrical equipment undergoes a maintenance and repair process, based on their condition, which is termed as preventive maintenance. The primary damages in transmission lines could be broken cables, damage to insulators, conductor corrosion and vibration damage; some of these defects are shown in Figure 1. Several methods have been employed to detect and analyze these anomalies. Geoffrey et al. presented the review of some of these methods to identify faults and classify their severity in power equipment using different image analysis approaches [1]. Also, Shawal et al. reviewed different methods for classifying the level of faults in electrical equipment [2].

In the past, the two most common methods of power equipment inspection were by foot patrol and helicopter-assisted investigation. Inspection based on foot patrolling is highly inaccurate, slow and is only for the surfaces of the power line equipment and therefore, more substantial defects can sometimes be overlooked. In helicopter-based inspection, the pilot flies the aircraft over the power lines while the camera operator films the conductors, insulators, pylons, and power transmission lines. Recently, Xie et al. presented a multiple-sensor platform method for overhead power line inspection based on the use of a large unmanned helicopter [3]. Although far faster in comparison to the foot patrol inspection, helicopter-assisted inspection is more expensive, hazardous and usually less accurate [4]. To overcome these problems, mobile and climbing robots came up as alternative solutions. The climbing robot travels along the conductors, achieving similar speed as in helicopter-assisted inspection. Recently, Qin et al. presented a method of autonomous inspection for transmission lines based on cable inspection robots [5]. In comparison to the foot patrolling, often requiring climbing on the power lines, the climbing robot is a safer and less time-consuming solution. Although having specific benefits, climbing robots are still not a practical solution to inspect the vast network of distribution lines [6]. Over the recent years, manned and unmanned aerial vehicles have been used for a broad spectrum of applications, supporting humans in dangerous and challenging environments, including the inspection and maintenance of power equipment. Advanced flight control techniques and image processing allow unmanned aerial vehicles (UAVs) to carry out fast inspection from some distance. Based on GPS data of both the UAV and electric towers, the embedded algorithms are able to perform the automatic tracking of power lines [6,7,8,9,10].

Compared with the conventional inspection methods, UAV-based inspection is more advanced, less expensive and safer. However, manned and unmanned aerial vehicles share some common problems, occurring while monitoring the power lines e.g., camera stabilization, pole and other obstacle tracking, etc. The acquired data, generally a sequence of images, are analyzed to assess the maintenance status of the power equipment. Also, the inspection by drones allows using different sensing payloads and, hence, to comprehensively inspect both power transmission lines and associated components using different types of sensors from an optimal point of view.

In the present work, we discuss and develop algorithms and methods for improving the capabilities of UAV-based inspection systems. Among the several targets which may be considered while monitoring the electrical infrastructure, we focus on temperature anomalies along cables, and on insulators hanging from electric towers. Regarding temperature anomalies, we perform multi-modal imaging integrating IR and visible data to identify faults and anomalies in power equipment. Regarding insulators, we use recursive CNN (Convolutional Neural Networks) to detect insulators in visible images and to classify their status. Section 2 describes the different approaches used to detect and classify insulators, and to analyse power transmission lines. Section 3 provides details about the experimentation and shows the results achieved.

## 2. Methods

The recent boost of UAV technology has increased the need of methods for object tracking and detection from RGB images, supporting the UAV intelligence or improving the functionalities of a real-time monitoring system based on UAV. Such methods should be robust and fast, and possibly should work simultaneously on the same data, in order to make the algorithm output more robust, for example with respect to noise (e.g., road lines vs power lines); or extremely fast, for example exploiting, pre-trained CNN models to detect specific components of the power equipment, namely, in our case, the insulators. Hence, in our research we aim at enhancing the scene understanding by implementing two processing modules, possibly to be embedded on drones: (i) one for the detection of power lines and hot spots, and improve its robustness by the integration of infrared (IR) and visible images; (ii) the other for the detection and classification of insulators, by implementing a CNN-based application able to perform the task in real time.

**Figure 2:**
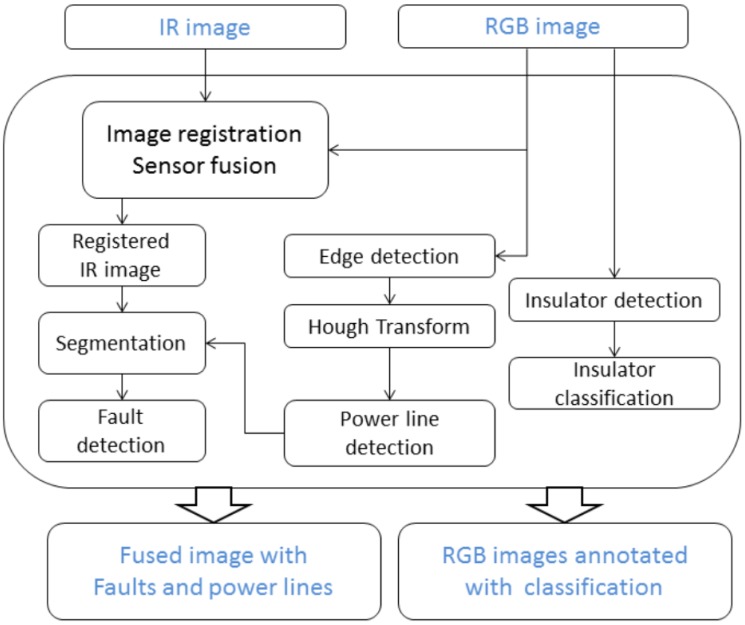
The processing pipeline.

In this work, we carried out the detection of hot spots and rusted insulators from sequences of images acquired by a drone surveying the electrical infrastructure. In order to detect hot spots, the first step is to identify power lines. We utilized visible images to extract power lines and rusted insulators, and IR images to identify hot spots. The processing pipeline is shown in Figure 2. In the following subsection, we provide a description of the methods developed to classify insulators from an optical video stream and to detect hot spots along with the power lines from visible and infrared images.

### 2.1. Insulators

In computer vision, the family of methods and algorithms developed for object recognition and classification has grown constantly in the last decades. Also, the recent achievements in machine learning and artificial intelligence have had a significant impact on the development of new tools and real-time applications. Therefore, the state-of-the-art literature recently provides several results of machine learning techniques applied to the detection, classification, and segmentation of electric towers. In [11], Sampedro et al. addressed the detection and classification tasks using a supervised machine learning approach; HOG features are used to train two multi-layer perception neural networks, one to predict whether the region inside an image is a tower (or not), and the other to distinguish the tower type on the basis of a training dataset.

In this work, we focus on the insulators, which are components of the electrical equipment and are essential to ensure the efficacy of the current conduction. Indeed, several researchers devoted quite an effort to develop methods to detect the insulators and classify them as damaged (an example is shown in Figure 1A,B) or normal. In [12], the author proposes an automatic method to detect insulators in images, based on image segmentation and template matching (using normalized cross-correlation). In [13] aerial images of power lines are used to test a method to detect insulators, based of the fusion of HOG and LBP features, reaching a correct detection of 89.1%. Zhao et al. in papers [14,15,16] documented an extensive study about the insulator detection and localization. At first, the authors proposed a method combining an orientation angle detection with a binary shape prior knowledge, and tested this method to complex aerial images, achieving the recognition of multiple insulators in real-time. More recently, Zhao et al. moved to a machine learning approach based on a multi-patch CNN feature extraction and classification, not only to localize insulators, but also to diagnose their condition (defect, normal). The results are excellent in accuracy, but it is also noted that the multi-patch strategy implies an increase of the computation burden. Also, Liu et al. in [17] proposed a method for the insulator localization based on deep learning, and achieved a true positive detection rate of 90.9%.

**Figure 3:**
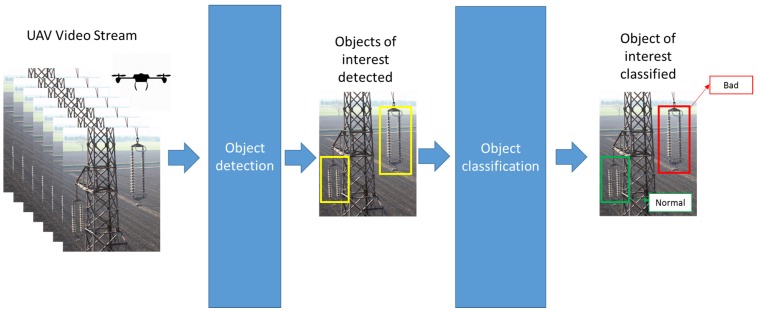
The scheme of the object detection and classification pipeline, applied to insulators.

The inspection of electrical components from an optical video stream is a standard task in computer vision, made of object detection and classification, generally addressed in two steps, as shown in Figure 3. First, a region proposal algorithm produces a set of candidate regions where the object is likely to be present. Then, the sub-image corresponding to the candidate region is handed to a classifier which produces in output a score about the presence of the object within the region. This approach has a key issue in finding the trade-off between the number of candidate regions produced and computing time. Also, the first step may be performed using a selective search approach (as in super-pixel-based methods [18]) or using a sliding windows approach, at multiple sizes and scales (e.g., [19]). It has been observed that the first step corresponding to a region proposal represents an important computational burden and is, indeed, the bottleneck towards fast detection. In our approach, in order to get an application able to provide automatic and fast classification of insulators, it was decided to resort to a particular kind of neural network architecture, named the Faster Region Convolutional Neural Network (Faster R-CNN) [20,21] that is an original region proposal network sharing features with the detection network that improves both region proposal quality and object detection accuracy. Faster R-CNN uses two networks: a deep fully convolutional network that proposes regions (named Region Proposal Network, RPN) and another module that classifies the proposed regions (classification network). The RPN produces region proposals more quickly than the selective search [22] algorithm used in previous solutions. By sharing information between the two networks, the accuracy is also improved, and this solution is currently the one with the best results in the latest object detection competitions. More precisely, the two sub-networks share the first layers which act globally as a deep feature extraction module. Several architectures can be used for building such a feature extraction module. Specifically, Inception Resnet v2 model was selected in this paper and instantiated for this particular application making use of TensorFlow [23]. Transfer learning was used to cope with the limited dataset of images, which is not sufficient for dealing with training from scratch. Namely, an inference graph for Inception Resnet v2 pre-trained on COCO dataset [24] has been imported. On the basis of the extracted feature, the RPN produces candidates for regions that might contain objects of interest. Namely, sliding a small window on the feature map, the RPN produces probabilities about the object presence in that region for region boxes of fixed aspect ratio and scale; a bounding box regressor also provides optimal size and position of the candidate rectangular areas in an intrinsic coordinate system. Candidates with a high probability of object presence are then passed to the classification network that is in charge of assessing the presence of an object category inside the region. As a training strategy, firstly, only the final fully connected layers of the two sub-networks were trained, leaving frozen all the other layers. In a fine-tuning phase, also the layers in the feature extraction module were optimized by using the training routines made available in TensorFlow. This architecture achieves, at the same time, region proposal (RP) and object detection. Faster R-CNN, designed for object detection in 2D images, is made of two networks: the former proposes candidate regions of interest, likely featuring an object inside (Region Proposals), and the latter uses the RPs to detect objects. Details about the built model, the results of the experimentation, and a description of the application deployed are reported in Section 3.

### 2.2. Power Lines

Focusing on the detection and tracking of power lines, Zhang et al. [10] extracted the power lines by applying thresholding to the gradient image. Similarly, Li et al. in [6] proposed a more complex filter based on a simplified pulse coupled neural network model. Candamo detected power transmission lines from low-quality videos combining the motion estimation at the pixel level with edge detection, followed by a windowed Hough transform [7]. A more comprehensive survey regarding computer vision methods used in the maintenance of power lines were given by Katransnik and Miralles in [4,9]. Hongwei et al. [25] had presented a fusion algorithm for the infrared and visible power lines image. Similarly, Larrauri et al. [26] identified areas of vegetation, trees and buildings close to power lines and calculated their distance from power lines. Simultaneously, the system processed the infrared images to detect hot spots in the power lines by estimating the threshold based on Otsu method and later to segment the lines from the background. On the other side, Lages et al. captured video streams from both infrared and visible cameras simultaneously [27]. Oliveira et al. discussed in detail the generation of hot spots in the transmission lines and later they had also used the same thresholding based segmentation to highlight hot spots [28]. Detecting power lines from a cluttered background is one of the most important and challenging tasks.

In general, all methods perform these two steps: (i) Identify expected power lines and remove the background. (ii) Inspect and identify possible faults from the power lines.

Our method follows the same scheme, performing these two steps, and each frame of the camera is processed individually. In more detail, our processing pipeline is described in the following:

- Infrared and visible image-based fusion.
- Generation of edge map using Canny edge detector.
- Hough transform to detect lines in the images.
- Extraction of power lines.
- Identifying faults by thresholding.

Even if the infrared imaging is a technique commonly used to test and inspect some components of the electrical equipment, such a technique may fail in the automatic detection of power lines, as the contrast between the background and power lines is usually not enough. Hence, we decided to apply multi-modal image processing, and to perform image fusion by registering both images, as in [25,29]. Image registration is a mapping between two or more images both spatially (geometrically) and with respect to intensity. Kim et al., presents a description and methods to perform registration [30]. Mathematically, registration is expressed as [31]:

(1) $$I_{2}=gI_{1}\left(f\left(x_{1};x_{2}\right)\right);$$

where $I_{1}$ and $I_{2}$ are two-dimensional images (indexed by $x_{1};x_{2}$), $f:(x_{1};x_{2})\mapsto (x_{1}^{\prime };x_{2}^{\prime })$ maps the indices of the distorted frame to match those of the reference frame, and *g* is intensity or radiometric transform.

The extraction of power lines is obtained through the following steps. After the contrast enhancement, the edge map is generated by applying the Canny edge detector [32]. Power lines having a sharp and linear profile are analysed using the Hough transform on the visible image. This way, linear objects are detected, and the set of lines includes edges of the roads, trees, poles, fences etc. [33]. The method usually parametrizes a line in Cartesian coordinates to a point in polar coordinates using the point-line duality equation:

(2) xcosθ+ysinθ=ρρ≥00≤θ≤π

where (x,y) is the point in the image plane in Cartesian coordinates. ρ is the perpendicular distance of the peak to the origin and θ correspond to the angle to the origin. We identified power lines and segmented that area from the image for further inspection. In the scene almost parallel segments, the Hough transform is complemented with a knowledge-based clustering in the Hough space in order to remove clutter and lines which are not power lines. Once the power lines are marked in the visible image, a mask is generated by segmenting power lines for better visualization and further inspection; the mask is applied to the IR image and, finally, the fault detection is obtained by thresholding. Various techniques have been used to classify anomalies in the infrared image of electrical equipment, and among those, the qualitative measurement is widely used. This qualitative measurement is based on the relative threshold criteria termed as comparative analysis. In order to extract the expected faults or hot spots within the IR image, we applied the Otsu’s thresholding method [27].

## 3. Experimentation and Results

The experimentation of this work has been carried out in the framework of the regional research project SCIADRO, which aims at developing the enabling technologies, crucial to accomplishing a rather rich and diverse span of missions through the use of a swarm of drones within a civilian scenario. The case study addressed in the project is to provide a tool to support the detection of the infrastructure components (power lines, towers, and insulators) and the diagnosis of their maintenance status simultaneously.

### 3.1. Data Acquisition

Infrared data and images in the visible spectrum were acquired near Parma (Italy) in November 2017. Data also include a small number of images containing defects, e.g., broken wires, rusted insulator and hot spots. The drone flew at a distance of approximately 10 mt from the power lines, and the visible and infrared cameras acquired simultaneously, at a low frame rate (about 5 f/s). The infrared camera is a FLIR A65sc with a standard lens (25 mm focal length H-FOV × V-FOV = 25° × 20°), while the visible camera is an industrial camera XIMEA 4.2 MP (square sensor). We acquired images to test on real data our method for the detection of the infrastructure and the diagnosis of its status. At this stage, two tasks have been implemented and tested: (i) classification of insulators as normal or rusted, using a convolutional neural network trained on our data; (ii) detection of power lines by image processing applied to RGB images.

### 3.2. Classification of Insulator

As explained in Section 2.1, the selected approach to insulator classification is Faster R-CNN, a network architecture which uses two sub-networks: a deep fully convolutional network that proposes regions (named Region Proposal Network, RPN) and another module that classifies the proposed regions (classification network). Training of the model was performed using the available dataset which is composed of 132 images, containing 160 distinct objects that are manually labelled. The dataset was then augmented applying both spatial and intensity transformations. With respect to other approaches that perform augmentation online directly during the training stage by applying transformations randomly, in this paper, augmentation was performed offline before training. Since the dataset contains a relatively small number of images and objects when compared to large general image datasets, there is no memory and efficiency concern in the present case.

A fraction corresponding to 80% of the dataset has been used as the training set, while the remaining images are used to evaluate the accuracy of the model in the classification task. The model for semantic image segmentation has been trained and fine tuned, achieving:

- Train accuracy = 100.0%
- Validation accuracy = 90.9%
- Final test accuracy = 97.3%

The hardware used for the training phase includes a NVIDIA GEForce GTX TITAN X on a PC with Intel octacore i7 and 16 GB of RAM.

Indeed, very few objects were classified incorrectly in our experimentation: (i) two not rusted glasses insulators recognized as rusted; (ii) one rusted ceramic or composite recognized as not rusted.

**Figure 4:**
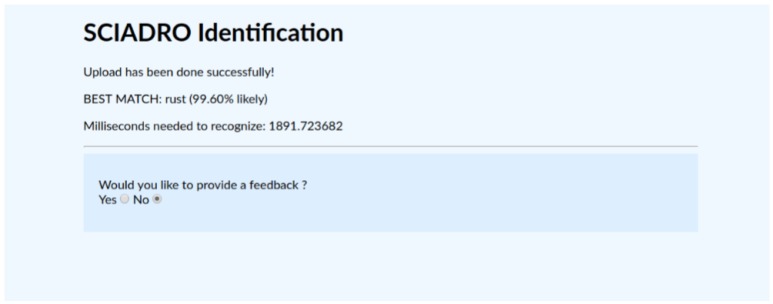
The web application layout resuming the processing output and performances.

**Figure 5:**
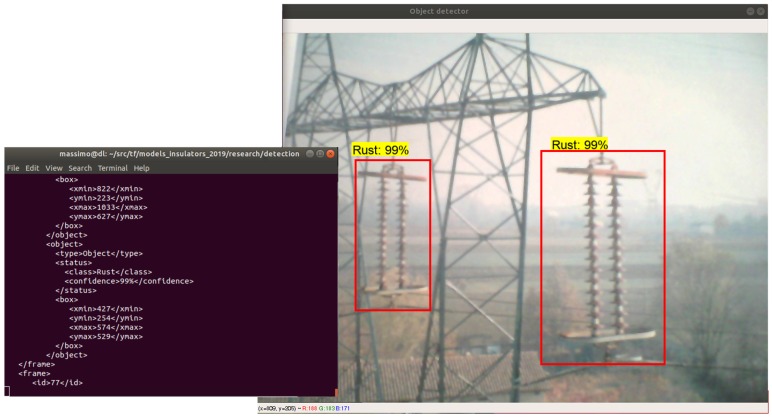
A screenshot of the output provided by the Python application.

Moreover, the model has been exploited to design and develop two applications. The first one is a Java Web application: the user may upload an image using a Web browser (e.g., Mozilla Firefox, Google Chrome) and receive the result of the classification, as shown in Figure 4; also, the user may select an image from a gallery and ask for a classification. The second application is a Python executable which reads a video, possibly captured in real-time by a camera, and processes it, storing the algorithm output in an XML file. The XML file (see Figure 5) counts the objects recognized, localizes each of them using the bounding box, and provides the classification confidence.

Results show a good performance, suitable for on board processing.

### 3.3. Power Lines Analysis

**Figure 6:**
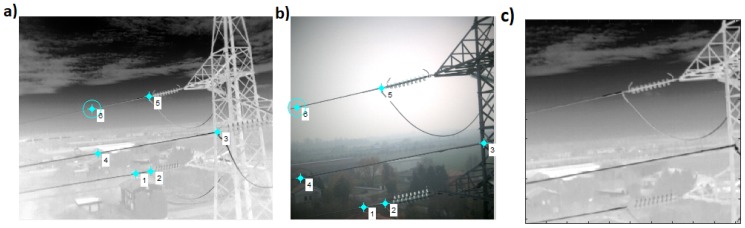
Image registration by user-defined control point selection to fuse multiple images from IR and visible cameras. We manually selected the significant feature from both IR and visible images which correspond to each other: (**a**) Control point selection from IR image. IR image has larger FOV (field of view), so we selected IR image to be registered; (**b**) Control point selection from Infrared image; (**c**) IR image after registration.

**Figure 7:**
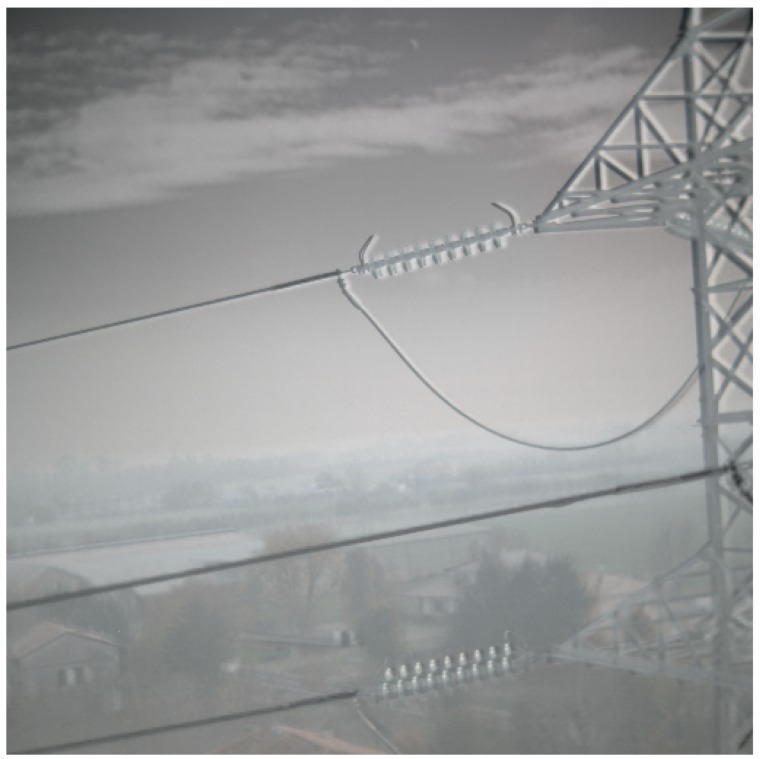
Overlapping of fixed (visible) and moving (IR) images with same size after registration.

The IR and visible cameras used in the experimentation have different fields of view and work in different wave bands; images were usually acquired from different viewpoints and in different operating conditions. Image registration was applied to pairs of IR and visible images, and the information was integrated into a single image in order to get a more effective identification of hot spots and defects in power equipment. In particular, the IR imaging of outdoor scenes in an uncontrolled environment poses significant challenges, because images show low contrast and high noise; also, in our experimentation the intensity value of most images is low. In addition to this, IR and visible pairs show low correlation in intensity and features which appear in the visible light image and may not appear in the infrared image. In order to overcome some of these issues and the influence of external environmental conditions, we employed a user-defined control point selection to fuse multiple images from IR and visible cameras. We manually selected the significant feature from both IR and visible images which correspond to each other as shown in Figure 6a,b. Since there is no radiometric distortion, the affine transformation is computed and applied as in [31]. Control point 1 in Figure 6a correspond to the control point 1 in Figure 6b and so on. IR camera (Figure 6a) captured the wider area of the field as compared to the visible camera (Figure 6b), therefore, we registered IR image with respect to the visible images as shown in Figure 6c. In order to see the difference between two images, the Overlay of two images is shown in Figure 7. We can observed that the two images are spatially aligned with no displacement.

**Figure 8:**
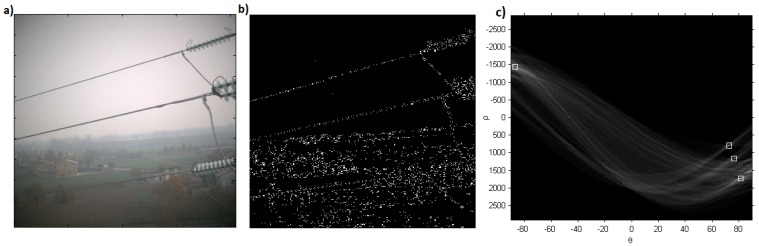
(**a**) Visible image of the tower and power cables; (**b**) Edges extracted from the visible image using Canny edge detector; (**c**) Detected peaks with Hough transform, where peaks correspond to the length of the line. ρ is the perpendicular distance of the peak to the origin and θ correspond to the angle. Occurrence of all positive angled peaks correspond to power lines.

The method described in Section 2.2 has been used to analyze the images acquired by a camera mounted on the drone flying close to the electric power lines. By way of example, an image is shown in Figure 8a. After the contrast enhancement, we applied the Canny edge detector to generate edge map [32] as shown in Figure 8: power lines along with sharp edges of background were detected. Power lines are almost parallel with approximately similar angle and distance from each other. By exploiting these properties, we improve the Hough transform by performing a knowledge-based line clustering in Hough space. This way segments not belonging to power lines are suppressed [6,10]. Figure 8c represents the detected peaks with Hough transform, where peaks correspond to the power transmission lines.

**Figure 9:**
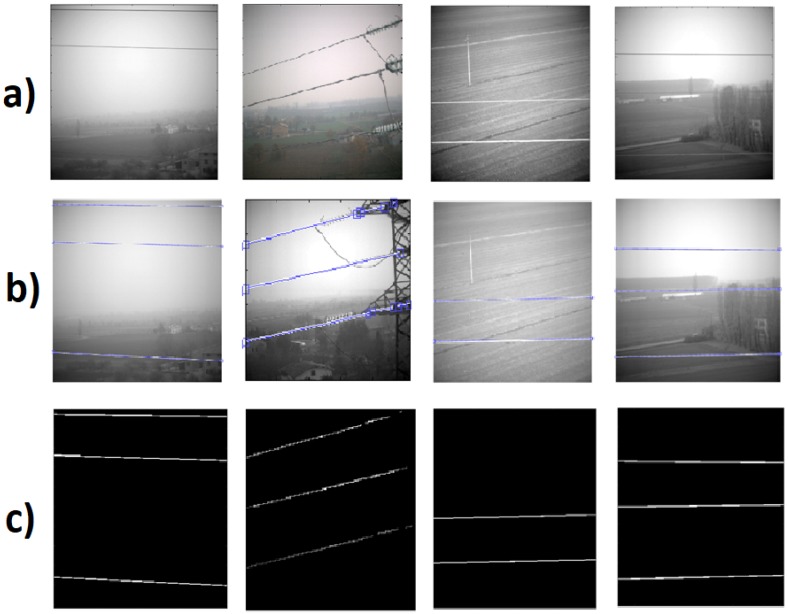
Example of power transmission lines with different backgrounds e.g., cluttered, semi-cluttered and with plain background: (**a**) Power lines on visible image. (**b**) Detected lines using Hough transform. (**c**) Segmented power transmission lines.

**Figure 10:**
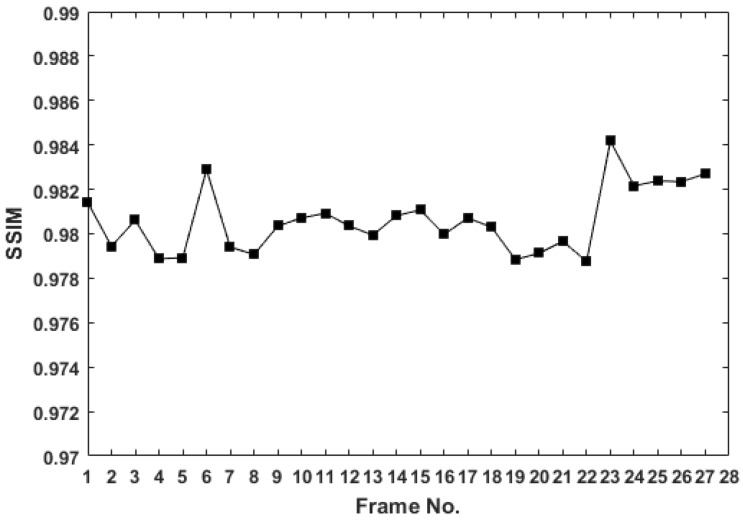
SSIM curve of detection results with reference to the ground truth on different frames of a video sequence with semi-cluttered background.

We applied the power line identification method to the data set acquired during acquisition campaign. Figure 9 presents power lines detection on some of the images. All detected lines are marked in the visible images and the power line mask is generated. It is important to note that the proposed method showed a good estimation of the power line in most of sequence of images taken from different perspectives and backgrounds, as shown in Figure 8. Different experiments have been conducted in order to validate the proposed method to detect power lines from aerial images. The set of images are taken from different perspectives of a power line in a continuous flight. In our results, manual reference segmentation drawn by experts are approximated as a ground truth. We define error as a mean error between ground truth and obtained detection, SSIM correspond to the structural similarity with reference to the ground truth. Execution time is the average time taken per image to detect lines. The Dice similarity coefficient (Dice_coeff_) was also used as a statistical validation metric to evaluate the performance of the method. Table 1 summarizes the results obtained on a single frame of different video sequence with different backgrounds. In almost all cases, the method performed well with minimum execution time. However, we observed better performance in the case of non-cluttered background where high SSIM and Dice coefficient correspond to the good segmentation of power lines. Figure 10 presents the SSIM curve on the different frames of a video sequence with semi-cluttered background. Horizontal axis shows the frame number, and vertical axis the obtained similarity index. It can be seen from the curve that the SSIM value remain in the same approximate range.

**Figure 11:**
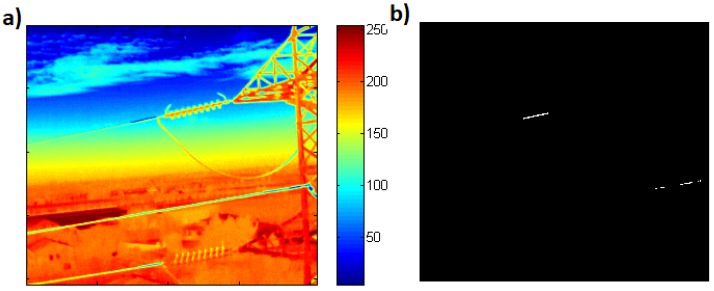
(**a**) Color map of IR image with semi-cluttered background, (**b**) Obtained hot spot in the power lines. After detecting power lines from visible image, histogram based thresholding on the registered IR image gives expected faults.

In order to extract the expected faults or hot spots within the IR image, we computed the histogram of an image, and performed Otsu’s thresholding to separate fault from the background as shown in Figure 11.

## 4. Discussion and Conclusions

Application of computer vision methods to analyze electrical faults and diagnose the condition of specific components of the infrastructure has been proved as one of the safer procedures for inspection. In the present work, we applied computer vision based methods on infrared and visible images to perform maintenance of power transmission lines and associated components.

We performed Hough transform based detection and IR imaging to detect possible faults in the power lines. Results, computed with different statistical parameters, compare the performance of obtained output with the manual ground truth. With an average of 0.98 similarity index, and 0.95 Dice similarity coefficient, in different background conditions, the method results effective for the detection of power lines.

Regarding the insulator detection task, our method achieved a performance at the state-of-the-art, but in addition, we provide a classification based on their status (pointing out even the presence of rust) in order to allow a predictive maintenance of the insulator chains, while most of the research is devoted to the insulator detection and localization, or limiting to shape anomalies (e.g., missing plates). We need to note that even the comparison with previous research is biased by the differences in data used for training the model; such data are in most cases not made available, difficult and costly to be collected. Also, an added value is to have implemented a Python application and a web service, in order to make it easier for operators to use the tool, and, on the other hand, to allow us to enlarge our dataset and improve further the performances and/or increase the interpretation of the insulator status.

Hence, we conclude from our results that the use of multi-modal imaging and of convolutional networks may improve the efficiency of drone surveying of the electrical infrastructure, and may open the way to the development of a novel monitoring payload (to be embedded in UAV) able to provide accurate and fast detection of faults and anomalies in power lines and insulators.

## Figures and Tables

**Table 1:** Obtained results on a single frame of different video sequence with cluttered and non-cluttered background.

Image	Error	SSIM	${\mathrm{Dice}}_{\mathrm{coeff}}$	Time (s)
Test 7 (Cluttered Background)	1.32	0.973	0.993	2.5
Test 6 (Non-cluttered Background)	0.98	0.997	0.994	3
Test 5 (Semi-cluttered Background)	0.44	0.971	0.972	3.1
Test 4 (Non-cluttered Background)	0.14	0.993	0.969	2.7
Test 3 (Non-cluttered Background)	0.14	0.976	0.954	3.9
